# *ALDH1A1* Gene Expression and Cellular Copper Levels between Low and Highly Metastatic Osteosarcoma Provide a Case for Novel Repurposing with Disulfiram and Copper

**DOI:** 10.1155/2022/7157507

**Published:** 2022-01-28

**Authors:** Jonathan B. Mandell, Nerone Douglas, Vrutika Ukani, Jan H. Beumer, Jianxia Guo, John Payne, Rebecca Newman, Luigi Mancinelli, Giuseppe Intini, Carolyn J. Anderson, Rebecca Watters, Kurt Weiss

**Affiliations:** ^1^Musculoskeletal Oncology Laboratory, Department of Orthopaedic Surgery, University of Pittsburgh, Pittsburgh, PA, USA; ^2^Department of Pharmaceutical Sciences, University of Pittsburgh, Pittsburgh, PA, USA; ^3^UPMC Hillman Cancer Center, Pittsburgh, PA, USA; ^4^Pittsburgh Veterinary Specialty and Emergency Center, Pittsburgh, PA, USA; ^5^University of Pittsburgh School of Dental Medicine, Department of Periodontics and Preventive Dentistry, Pittsburgh, PA, USA; ^6^Department of Medicine, University of Pittsburgh, Pittsburgh, PA, USA; ^7^Departments of Chemistry and Radiology, University of Missouri, Columbia, MO 65211, USA; ^8^Departments of Anatomic Pathology and General Surgical Oncology, University of Pittsburgh, Pittsburgh, PA, USA

## Abstract

Aldehyde dehydrogenase 1A1 (ALDH) is a cancer stem cell marker highly expressed in metastatic cells. Disulfiram (Dis) is an FDA-approved antialcoholism drug that inhibits ALDH and has been studied as a candidate for drug repurposing in multiple neoplasia. Dis cytotoxicity in cancer cells has been shown to be copper-dependent, in part due to Dis's ability to function as a bivalent metal ion chelator of copper (Cu). The objectives of this research were to test *ALDH* expression levels and Cu concentrations in sarcoma patient tumors and human osteosarcoma (OS) cell lines with differing metastatic phenotypes. We also sought to evaluate Dis + Cu combination therapy in human OS cells. Intracellular Cu was inversely proportional to the metastatic phenotype in human OS cell lines (SaOS2 > LM2 > LM7). Nonmetastatic human sarcoma tumors demonstrated increased Cu concentrations compared with metastatic tumors. qPCR demonstrated that *ALDH* expression was significantly increased in highly metastatic LM2 and LM7 human OS cell lines compared with low metastatic SaOS2. Tumor cells from sarcoma patients with metastatic disease displayed significantly increased *ALDH* expression compared with tumor cells from patients without metastatic disease. Serum Cu concentration in canine OS versus normal canine patients demonstrated similar trends. Dis demonstrated selective cytotoxicity compared with human multipotential stromal cells (MSCs): Dis-treated OS cells demonstrated increased apoptosis, whereas MSCs did not. CuCl_2_ combined with Dis and low-dose doxorubicin resulted in a superior cytotoxic effect in both SaOS2 and LM7 cell lines. In summary, *ALDH* gene expression and Cu levels are altered between low and highly metastatic human OS cells, canine samples, and patient tumors. Our findings support the feasibility of a repurposed drug strategy for Dis and Cu in combination with low-dose anthracycline to specifically target metastatic OS cells.

## 1. Introduction

Sarcoma encompasses a heterogeneous group of mesenchymal neoplasia whose prognoses mainly depend on the presence or absence of metastatic disease [1, 2]. Osteosarcoma (OS) is the most common primary neoplasm of bone and is the third leading cause of cancer-related death in patients under the age of 20 [3, 4]. OS most commonly originates in the extremities (distal femur, proximal tibia, and proximal humerus) and preferentially metastasizes to the lungs [5]. Patients with OS pulmonary metastases have especially poor prognoses with survival rates of 15–30%. These suboptimal outcomes have persisted for over 30 years [5–9].

OS patients currently receive multiagent chemotherapy including doxorubicin (Dox), an anthracycline known to cause severe myelosuppression and long-term cardiotoxicity [10–12]. The identification and testing of novel targets that are targeted to metastatic OS biology has been our main area of investigation, but many previous studies from our group have been performed with murine OS cells in culture and animal models [13–17].

Aldehyde dehydrogenase 1A1 (ALDH) is a stem cell- and cancer stem cell-associated factor that has been studied extensively for its role in cancer biology [18–22]. ALDH belongs to a superfamily of enzymes that are responsible for metabolizing aldehydes into carboxylic acids and enabling cancer cells to resist the oxidative stress imparted by chemotherapeutic agents [23–26]. Disulfiram (Dis) is an FDA-approved antialcoholism drug, which can act as an inhibitor of ALDH activity and has been extensively studied as an anticancer therapy. Several basic and clinical studies suggest that the addition of copper (Cu) compounds enhances Dis's antineoplastic potency [27–33]. Despite extensive research on Dis repurposing, the relationship between ALDH, Cu, and Dis and how they contribute to the efficacy of Dis + Cu treatment for cancer patients has not been explored in sarcomas.

Although rare in humans, OS is seen frequently in dogs, where they invariably demonstrate a rapid progression to metastatic disease and death [34, 35]. In this study, we investigated differences in *ALDH* expression and serum Cu concentrations in human and canine sarcoma clinical samples from patients in our practices with known metastatic histories. Based on our previous work [17], we hypothesized that human OS cells and clinical specimens with differing metastatic phenotypes would demonstrate predictable differences in *ALDH* gene expression and Cu concentration levels. We tested the ability of Cu to alter the potency of Dis treatment in human OS cells, as well as the potential of Dis + Cu to reduce the required cytotoxic dose of Dox. We predicted that Cu would alter the susceptibility of human OS cells to Dis-mediated toxicity and that the combination of Dis + Cu might enable cytotoxicity with lower Dox doses.

## 2. Materials and Methods

### 2.1. Cell Culture

The SaOS2 human OS cell line was purchased from American Type Culture Collection (ATCC). LM2 and LM7 cell lines were generously provided by Dr. Eugenie Kleinerman in the Pediatric Oncology Division at MD Anderson after in-house genomic authentication. SaOS2 is the parental cell line of the LM2 and LM7 cell lines and has low metastatic potential. Briefly, to generate the LM2 and LM7 cell lines, SaOS2 cells were injected into the tail veins of nude mice and spontaneous metastases were collected. Metastatic cells were isolated and cycled through the lungs of experimental animals twice and seven times to yield the highly metastatic LM2 and LM7 cell lines, respectively [36]. All human OS cell lines were grown and maintained using identical culture media and conditions. Cells were grown in T175 flasks (Corning) with culture media of DMEM (Corning) supplemented with 10% FBS (Gibco), 1% penicillin/streptomycin Ab solution (Gibco), 1% MEM nonessential amino acids solution (Gibco), and 1% vitamin solution 100X (Gibco) at 37°C in a humidified incubator.

Human multipotential stromal cells (MSCs, Texas A&M Institute for Regenerative Medicine) were plated at 2 × 10^4^ cells per cm^2^ in *α*-MEM (Gibco) supplemented with 16.5% fetal bovine serum not-heat inactivated (Atlanta Biologicals), 2 mM L-glutamine (Gibco), 100 units/mL penicillin G, and 100 *µ*g/mL streptomycin sulfate (Gibco) at 37°C with 5% CO_2_. 24 hours after plating, as indicated, 12.5/25 *µ*M of Dis or an equal volume of the appropriate vehicle (dimethyl sulfoxide Sigma-Aldrich) was added to the cells in the growth medium at 24 and 48 hours.

### 2.2. Patient Samples for Cu Analysis

Human patient sarcoma tumors, blood, and tumor-derived cells were obtained from our IRB-approved Musculoskeletal Oncology Tumor Registry and Tissue Bank (STUDY20010034) that is supplied by the senior author (kW) and his clinical partners. The clinical history of each patient was obtained from electronic medical records and patient information was deidentified. Human sarcoma samples were processed in a sterile hood which involved antibiotic/antimycotic treatment followed by a wash with PBS. Specimens were mechanically minced, flash frozen using liquid nitrogen, and stored at −80°C. Canine blood samples were obtained from the Pittsburgh Veterinary Specialty and Emergency Center (PVSEC). Human and canine blood samples were collected in Streck tubes and centrifuged for 2k RPM for 10 minutes. Blood plasma was collected from the supernatant, aliquoted, and stored at −80°C.

### 2.3. Atomic Absorption (AA) Spectrophotometry Cu Analysis

Human OS cell lines, human sarcoma samples, human blood plasma, and canine blood plasma were processed and stored as described above and Cu concentrations were determined using a Perkin Elmer AAnalyst 600 atomic absorption spectrophotometer adjusted to detect Cu (324.8 nm) as previously described [17]. The final concentration of Cu in blood plasma was calculated as total *µ*g/ml. Intracellular and tumor Cu concentrations were calculated as (final sample concentration of Cu (ng/mL))/(total sample protein concentration (mg/ml)) and recorded as ng Cu/mg protein.

### 2.4. Patient Tumor Cells for qPCR

De-identified OS, chondrosarcoma, leiomyosarcoma, rhabdomyosarcoma, and high-grade sarcoma patient tumor samples were washed twice with 3x penicillin/streptomycin solution diluted in sterile PBS and then *washed* once with sterile PBS. Under sterile tissue culture conditions, tumors were placed in sterile Petri *dishes* and minced with sterile forceps and surgical scissors. Equal amounts of dispase (ThermoFisher) at 2 mg/ml and collagenase XI (Sigma) at 2.4 units/ml were added to minced samples and incubated for 90 minutes, shaking the dishes every 15 minutes. After centrifugation to remove the enzymes, digested tumor cells were cultured in DMEM (ThermoFisher) with 10% FBS (Gibco) and 1% penicillin/streptomycin.

### 2.5. Quantitative PCR

mRNA was collected from SaOS2, LM2, and LM7 cell lines and patient tumor cells using the RNeasy Kit (Qiagen), and cDNA was obtained using the Reverse Transcriptase Kit (Applied Biosystems). qPCR was performed using SYBR Green Supermix (BioRad). GAPDH, SYMPK, ANKRD, and HPRT reference genes were selected as housekeeping genes. Delta CT values for OS cell lines (SaOS2, LM2, and LM7) were calculated using the geometric mean of the three selected housekeeper genes GAPDH, SYMPK, and ANKRD. Delta CT values for patient derived tumor cells were calculated using HPRT housekeeper gene. Differential gene expression between OS cell lines and patient derived tumor cells was determined for *ALDH*.

### 2.6. Cell Viability Assays

SaOS2 and LM7 were plated in black-walled 96 well plates (10,000 cells) and cultured for 24 hours. Media was replaced with culture media with fold dilutions of Dox, CuCl_2_, and Dis (98 nM–50 *µ*M). Additionally, Dis fold dilutions were tested in combination with fixed concentrations of CuCl_2_ (50, 250, and 500 nM). 24 hours after drug addition, cells in each well were stained (10 *µ*L) with a mammalian PrestoBlue Cell Viability Reagent (Invitrogen). PrestoBlue cell viability reagent-stained cells were incubated for 20 min at 37°C. The fluorescence intensity was evaluated using a FluoroMax®-4 spectrofluorometer, with excitation and emission wavelengths of 590/560 nm, respectively.

### 2.7. Cell Cytotoxicity and Recovery Assays

SaOS2 and LM7 were plated (0.1 × 10^6^) in 6-well plates and cultured for 24 hours. Cells were treated over 24 hours with Dox (0.05–2.5 *μ*M), Dis (0.05–2.5 *μ*M) + CuCl_2_ (0.2 *μ*M), or Dis (0.05–2.5 *μ*M) + CuCl_2_ (0.2 *μ*M) + Dox (0.1 *μ*M). Cells were washed with PBS, treated with 100 *μ*L TrypLE Express, and resuspended in culture media. Viable cell counts were obtained using a Trypan Blue exclusion assay and an automated hemocytometer (Bio-Rad). Treated human OS cell lines were all recultured with fresh media without drugs in 6-well plates and monitored for cellular growth and recovery of cells for two weeks.

### 2.8. MTT Cell Cytotoxicity Assay

2 × 10^4^ cells per well were plated in 96-well *plates* and after 24 hours, they were treated with Disulfiram at the indicated concentrations. After 24 and 48 hours, MTT assay was performed according to the manufacturer's protocol (Abcam). Optical density at 590 nm was used as a measure of proliferation and the percentage of cytotoxicity was calculated with the following equation, using corrected absorbance:

(1) $$\begin{array}{c} \%\text{cytotoxicity}=\frac{\left(100\times \left(\text{control}-\text{sample}\right)\right)}{\text{control}}. \end{array}$$

### 2.9. Statistics

All statistical methods were performed using Prism 9.0 (GraphPad, La Jolla CA). Multiple groups were compared using an ordinary one-way ANOVA. Two groups were compared using an unpaired *t*-test. In all cases, *p* < 0.05 (^*∗*^) and *p* < 0.01 (^*∗∗*^) were considered significant, as indicated in each figure.

## 3. Results

Highly metastatic human OS cell lines and patient tumor samples display decreased Cu levels compared with less metastatic samples.

**Figure 1:**
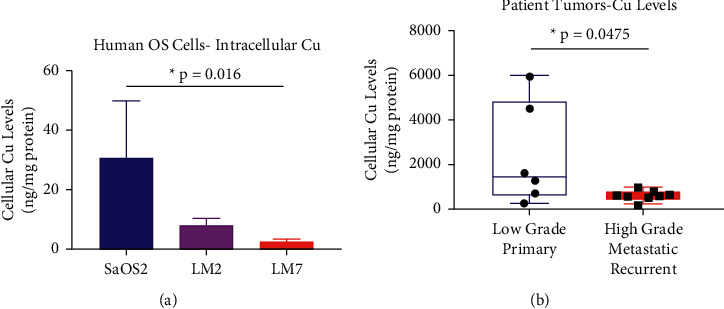
Metastatic OS cell lines and patient sarcomas display lower levels of intracellular Cu compared with nonmetastatic samples. (a) Human OS cell line triplicates (SaOS2, LM2, and LM7) were cultured under identical conditions and then collected for protein quantification and intracellular Cu analysis using atomic absorption spectrophotometry (AA). LM2 and LM7, which demonstrate increased metastatic potential, displayed significant decreases in intracellular Cu compared with parental SaOS2. (b) Deidentified patient information was used to separate sarcomas, including OS, that came from patients with metastatic disease and those with no signs of metastatic disease (*n* = 14). Tumors from patients with metastatic disease displayed significantly decreased Cu levels compared with tumors from nonmetastatic patients. The statistical significance of differences among the cell types was assessed using ordinary one-way ANOVA, while the difference between the two patient tumor groups was assessed using two-tailed Student's unpaired *t*-test. Data represent mean ± SD: ^*∗*^*p* < 0.05, ^*∗∗*^*p* < 0.01, and ^*∗∗∗*^*p* < 0.001.

Intracellular Cu levels were significantly increased (^*∗*^*p* = 0.016) in parental SaOS2 cells compared with highly metastatic LM7 cells (Figure 1(a)). We also assessed the differences in Cu levels in tumors from sarcoma patient tumors with differing metastatic histories. Tumor samples from sarcoma patients, including OS, who developed metastatic disease demonstrated significantly lower (^*∗*^*p* = 0.048) endogenous levels of Cu compared with samples from patients who did not develop metastases (Figure 1(b)). This indicates that intracellular Cu concentration is inversely proportional to metastatic phenotype in human cell lines and patient tumors.

### 3.1. Blood Plasma of Human and Canine Patients with Metastatic Sarcomas Display Increased Cu Levels

**Figure 2:**
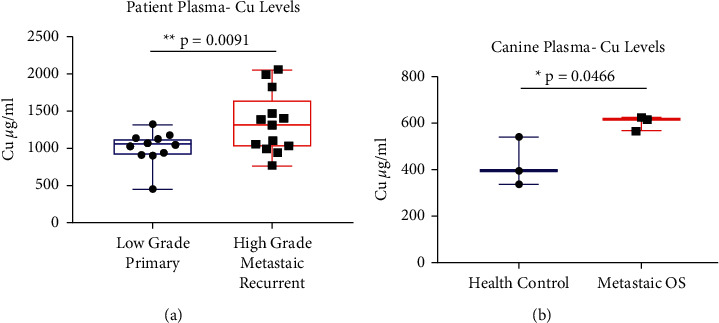
Blood plasma from humans and canines with metastatic sarcomas display significantly increased Cu levels. (a) Blood plasma samples from human sarcoma, including OS patients with metastatic disease, display significantly increased serum Cu levels compared with patients without metastatic disease (*n* = 24). (b) Blood plasma samples from canine OS patients display significantly increased Cu levels compared with plasma from healthy control canines (*n* = 6). The statistical significance difference between the two groups were assessed using two-tailed Student's unpaired *t*-test. Data represent mean ± SD: ^*∗*^*p* < 0.05, ^*∗∗*^*p* < 0.01, and ^*∗∗∗*^*p* < 0.001.

Blood plasma quantitation demonstrated that Cu was significantly increased (^*∗∗*^*p* = 0.009) in the sera of patients with metastatic human sarcoma (which includes OS) compared with patients who did not metastasize (Figure 2(a)). Blood plasma from canine OS patients, which invariably display a rapid progression to metastatic disease, showed significantly increased Cu (^*∗*^*p* = 0.047) compared with healthy control canine blood plasma (Figure 2(b)). These increased Cu levels in plasma of both human and canine patients may be correlated with metastatic disease progression.

### 3.2. Highly Metastatic Cell Lines and Patient Tumor Cells Display Increased *ALDH* Gene Expression

**Figure 3:**
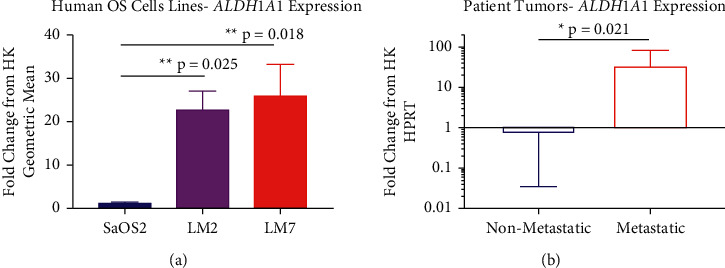
Metastatic OS cell lines and patient tumors display increased *ALDH* expression compared with nonmetastatic samples. (a) *ALDH* gene expression was significantly increased in both LM2 and LM7 compared with SaOS2 cells. (b) In human patient-derived tumor cells, *ALDH* gene expression was significantly increased in tumor cells from patients with metastatic disease compared to patients without metastatic disease (*n* = 14). The statistical significance of differences among the treatment was assessed using ordinary one-way ANOVA, while the difference between metastatic and nonmetastatic was assessed using two-tailed Student's unpaired *t*-test. Data represent mean ± SD: ^*∗*^*p* < 0.05, ^*∗∗*^*p* < 0.01, and ^*∗∗∗*^*p* < 0.001.

We performed quantitative PCR to compare gene expression level differences of *ALDH* in human OS cell lines and primary patient samples. qPCR displayed significantly higher gene expression of *ALDH* in highly metastatic LM2 cells (^*∗∗*^*p* = 0.025) and LM7 cells (^*∗∗*^*p* = 0.018) compared with less metastatic SaOS2 cells (Figure 3(a)). We also observed that tumor cells derived from patients with metastatic disease displayed significantly increased *ALDH* gene expression (^*∗*^*p* = 0.021) compared with tumor cell from patients with no evidence of metastatic disease (Figure 3(b)). These findings are supported by our previous investigations in which we observed increased *ALDH* expression in highly metastatic OS cell lines [14, 16, 17] as well as increased ALDH enzymatic activity in human OS cell lines and bone sarcoma patient samples [15, 37].

### 3.3. Dis Treatment Alters the Viability of OS Cells but Not Human Multipotential Stromal Cells (MSCs)

**Figure 4:**
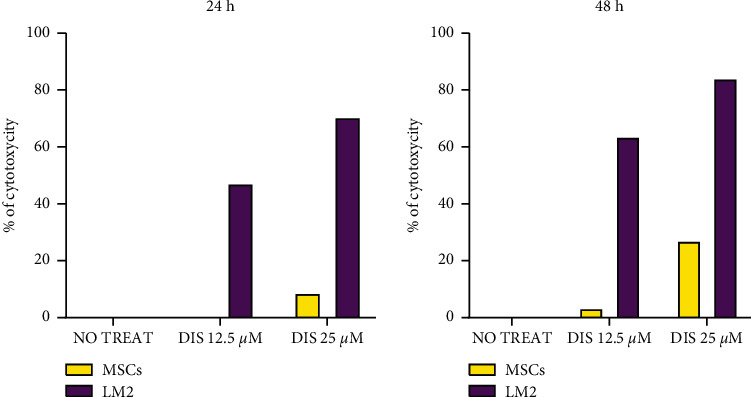
Selective cytotoxicity of Dis for metastatic OS cells compared with MSCs. After 24 h and 48 h of Dis treatment at the indicated concentrations, cytotoxicity was determined by MTT assay in LM2 cells (red bars) and MSCs (blue bars). LM2 cells were much more sensitive to the cytotoxic effects of Dis than MSCs. Dis treatment causes changes in apoptosis-related genes in OS cells but not human multipotential stromal cells (MSCs).

To investigate if Dis-associated toxicity demonstrated selectivity for metastatic OS cells, MTT assays were performed after 24 and 48 hours of Dis treatment (12.5 mM and 25 mM) on LM2 cells and MSCs. As shown in Figure 4, Dis treatment resulted in much greater cytotoxicity in LM2 cells than MSCs at both Dis doses, suggesting that Dis is selectively cytotoxic to cancer cells.

**Figure 5:**
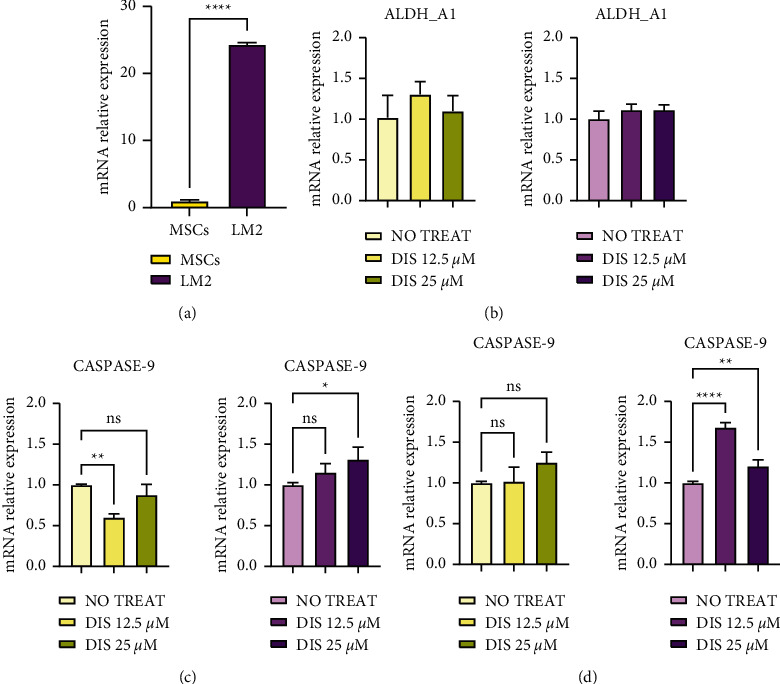
Dis treatment causes changes in apoptosis-related genes in OS cells but not human multipotential stromal cells (MSCs). After 4 h of Disulfiram (Dis) treatment at the concentration of 12.5 and 25 *µ*M, *ALDH*, caspase 9, and caspase 3 expressions were checked by qRT-PCR in LM2 cells (red bars) and MSCs (blue bars). (a) Untreated LM2 cells expressed significantly greater *ALDH* expression compared with mesenchymal stem cells (MSCs, *n* = 3). (b) *ALDH* expression after 4 h of DIS treatment (*n* = 3). (c) Caspase 9 gene expression after 4 h of Dis treatment (*n* = 3). hPPIA was used as a housekeeping gene. The statistical significance of differences among the treatment was assessed using ordinary one-way ANOVA with Dunnett's multiple comparisons test, while the difference between the two cell types was assessed using two-tailed Student's unpaired *t*-test. Data represent mean ± SD: ^*∗*^*p* < 0.05, ^*∗∗*^*p* < 0.01, and ^*∗∗∗∗*^*p* < 0.0001.

**Figure 6:**
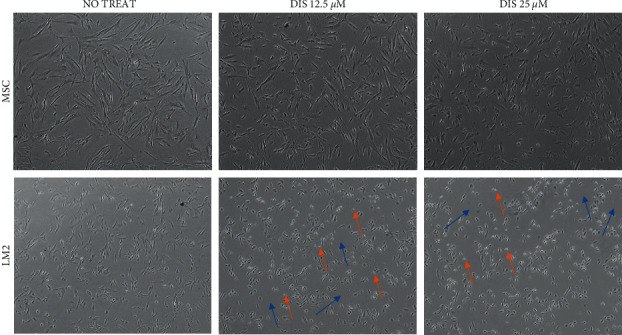
Dis treatment causes visible distress in LM2 cells but not MSCs. Representative images of both MSC and LM2 cells after 4 hours of treatment with Dis at the concentrations of 12.5 *µ*M and 25 *µ*M. LM2 appear smaller and display unhealthy signs like more cellular debris (blue arrows) and round dots inside which are probably apoptotic bodies (orange arrows). MSCs, even at high Dis concentration, remain healthy appearing.

To further uncover the mechanism through which Dis mediates OS cell toxicity, we investigated if the harmful effects that we observed in LM2 OS cells and not in MSCs (Figure 4) could also be due to alteration of *ALDH* expression levels. To this purpose, we isolated RNA from both LM2 and MSCs after Dis treatment and evaluated *ALDH* expression. At baseline (without treatment), LM2 cells expressed dramatically more ALDH than MSCs (Figures 5(a) and 5(b)). Due to the strong toxic effects of Dis, we treated the cells for 4 hours to obtain sufficient RNA for subsequent analysis. After 4 hours of treatment, LM2 cells demonstrated obvious distress, while MSCs did not (Figure 6). Given the presence of distress signs in LM2, we quantified the expression of apoptosis-related genes and observed a statistically significant increase of both caspases 3 and 9 in LM2 cells but not in MSCs after 4 h of Dis treatment (Figures 5(c) and 5(d)). There was no significant difference in *ALDH* expression after Dis treatment (Figure 5(b)). Together, these data suggest that the Dis exposure causes premature apoptosis of LM2 cells but not of MSCs and this selectivity of action may be due to the inherent differences in *ALDH* expression between malignant and nonmalignant cells.

### 3.4. Dis and CuCl_2_ Treatment Results in Decreased Cell Viability of LM7 Compared with Dis Monotreatment

**Figure 7:**
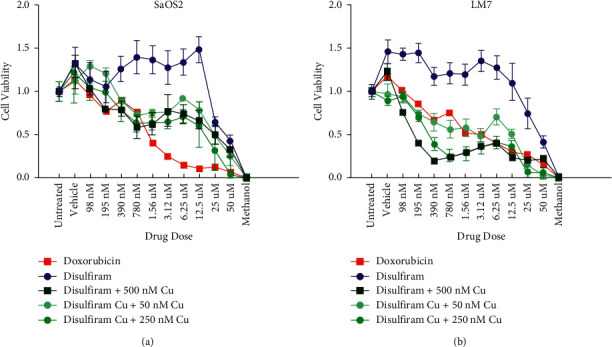
Cu potentiated cytotoxicity of Dis reduces cell viability in both SaOS2 and highly metastatic LM7 cell lines. (a) SaOS2 (*n* = 6) and (b) LM7 (*n* = 6) cells were subjected to fold dilutions of Dox, Dis + CuCl_2_, or Dis monotherapy over 24 hours. After treatment, cells were stained with PrestoBlue viability stain, and fluorescence intensity was evaluated to measure cell viability. The addition of CuCl_2_ significantly decreased the cell viability in both LM7 and SaOS2 compared to monotherapy of Dis. Dis + Cu combination treatment resulted in increased cytotoxicity to LM7 compared to Dox monotherapy.

Given the significant upregulation of *ALDH* expression in LM7, as well as the retention of intracellular Cu levels in SaOS2, we tested Cu-potentiated Dis cytotoxicity in both OS cell lines over 24 hours. SaOS2 treated with Dox displayed a 50% reduction in cell viability (IC_50_) at 1.2 *µ*M, and Dis displayed an IC_50_ at 25 *µ*M (Figure 7(a)). LM7 treated with Dox displayed an IC_50_ at 2.5 *µ*M, and Dis displayed an IC_50_ at 25 *µ*M (Figure 7(b)). The addition of varying concentrations of CuCl_2_ supplement (50 nM, 250 nM, and 500 nM) resulted in potentiation of Dis and increased cytotoxicity in both SaOS2 and LM7 compared with Dis monotherapy. Interestingly, CuCl_2_ addition to Dis resulted in greater cytotoxicity of LM7 (IC_50_ = 0.6 *µ*M) compared to that of SaOS2 (IC_50_ = 1.2 *µ*M).

### 3.5. Dis + CuCl_2_ Requires Low-Dose Dox to Effectively Kill and Prevent Recovery of LM7 *In Vitro*

**Figure 8:**
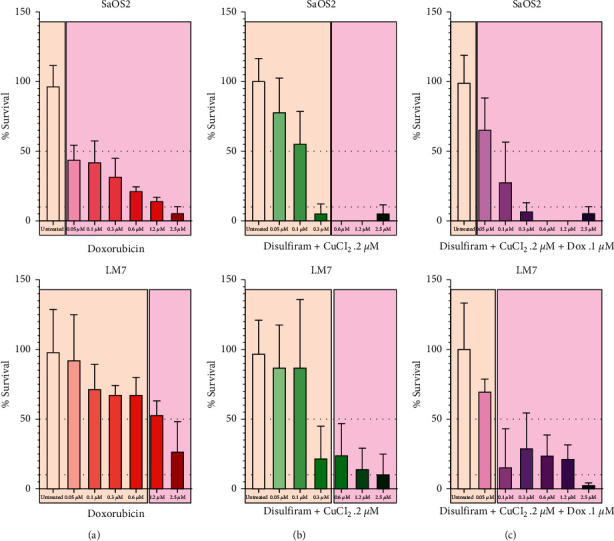
Combination treatment of Dox, Dis, and CuCl_2_ effectively reduces viability of human OS cells and eliminates recovery *in vitro*. Viable cell counts of SaOS2 and LM7 were obtained using a Trypan Blue exclusion assay and a hemocytometer (colored bar graphs). After 24 h of treatment exposure, treated human OS cell lines were all recultured with fresh media without drug and monitored for recovery of cells; tan background shading indicates cells were able to grow after drug removal, and pink background shading indicates cells were unable to grow. (a) SaOS2 treated with Dox were unable to recover after a .05 *µ*M dose, while LM7 was unable to recover after 1.2 *µ*M. (b) SaOS2 and LM7 given Dis + CuCl_2_ were both unable to recover after a 0.6 *µ*M Dis dose. (c) LM7 given triple treatment with 0.1 *μ*M Dis, 0.2 *μ*M CuCl_2_, and 0.1 *μ*M Dox was unable to recover after drug removal.

To assess the long-term treatment effects on cytotoxicity and cellular recovery of human OS cells, SaOS2 and LM7 were treated for 24 hours with monotherapies and combination treatments of Dox, Dis, and CuCl_2_. After viable cell counts were obtained, cells were recultured in media without drugs, and cellular growth was assessed over 2 weeks. OS cell lines treated with 0.1 *μ*M Dox demonstrated 50% survival but were able to recover after drug removal. Dox treatment was highly cytotoxic to SaOS2 with cells unable to recover after only a 0.05 *µ*M dose, while LM7 required 1.2 *µ*M Dox to achieve this (Figure 8(a)). When administered with 0.2 *µ*M CuCl_2_, Dis was able to render both SaOS2 and LM7 cells unable to recover after a 0.6 *µ*M dose (Figure 8(b)). “Triple treatment” with 0.1 *μ*M Dis, 0.2 *μ*M CuCl_2_, and 0.1 *μ*M Dox was highly cytotoxic to metastatic LM7, and these cells were unable to recover after drug removal (Figure 8(c)). This demonstrates that a low dose of Dox combined with Dis + CuCl_2_ may treat highly metastatic human OS cell lines and prevent cellular recovery after drug removal.

## 4. Discussion

We investigated *ALDH* gene expression and endogenous Cu levels in human OS cell lines, canine and human serum, and patient-derived tumors of differing metastatic potentials and clinical metastatic histories. Our findings demonstrate that low metastatic SaOS2 cells, tumors from patients without metastatic disease, and sera from dogs with OS had significantly greater endogenous intracellular Cu compared with highly metastatic LM2 and LM7 cells, metastatic tumors, and the sera of healthy dogs. These data are consistent with previous results from our group that reported an animal model using murine OS cells (K12 and K7M2) [17].

Metastatic sarcoma cells from patient samples demonstrated increased *ALDH* expression compared with patients without evidence of metastasis. We also evaluated the ability of Cu to affect the cytotoxicity of Dis and found that combination treatment could produce a durable, cytotoxic effect on the highly metastatic human OS cell line, LM7. Treatment with Dox, Dis, and Cu produced significantly greater cytotoxicity and inhibited growth recovery by highly metastatic LM7 cells.

The use of Dox chemotherapy has been the mainstay in OS treatment and facilitated a dramatic improvement compared with surgery alone [38, 39]. However, the dose-dependent cardiotoxic effects of Dox have been well documented and pose difficulties for OS patients, their families, and clinicians [40]. Faced with this problem, we assessed the ability of human OS cell lines to recover after “triple treatment” with low-dose Dox along with Dis and Cu. We demonstrated that although Dox monotherapy effectively reduced metastatic OS cell viability, these cells could effectively recover after drug removal in culture. Interestingly, Dis + Cu combination treatment resulted in a significant reduction in human OS cell viability, but these cells also eventually recovered. Only our “triple treatment” regimen of low-dose Dox, Dis, and Cu demonstrated both initial cytotoxicity and the inability of cells to recover. The potential to deliver agents that specifically target metastatic OS cells while reducing the required dose of Dox is noteworthy.

Dis repurposing for cancer treatment has been proposed for decades and is currently believed to have myriad chemotherapy sensitizing, cytotoxic, and antimetastatic effects on cancer cells [41, 42]. Dis is well-known as an inhibitor of ALDH and was originally utilized clinically as part of aversion therapy for chronic alcoholism [43]. ALDH is also widely appreciated to be a cancer stem cell marker present in many types of neoplasia, including sarcoma [44, 45]. Increased ALDH activity has been linked to cancer cell survival and increased resistivity to drug therapies, which was once thought to be the main anticancer effect of Dis [22, 46]. Dis has been shown to act as a strong Cu chelator, and Dis + Cu treatment resulted in a potentiated cytotoxic effect, as well as potent sensitization to originally resistant chemotherapeutics such as Dox [47]. The direct mechanism of how Cu chelating compounds act on cancer cells is still incompletely understood [27]. Sufficient responses against cancer cells have been found to be due to generation of reactive oxygen species during Cu chelation [32], as well as pharmacological activity of the drug metabolite after chelation [33]. Proteasome inhibition has been shown using a combination therapy of thiocarbamate drugs and Cu compounds in leukemia and breast cancer cells [48–50]. More recent studies have shown that Dis + Cu acts upstream of proteasome activity by binding to NPL4 subunit on p97 segregase [51] and is independent of ALDH inhibition [52]. Importantly, the main cytotoxic effects elicited by Dis were found to be due to the active metabolite (DDC) chelated with Cu^2+^ (DDC-Cu). Although applicable to all cancers being studied for Dis repurposing, a significant amount of the mechanistic testing into DDC-Cu mechanism of action was performed in U2OS, another well-known human OS cell line [51]. This further supports the rationale of studying Dis and Cu treatment in treatment-refractory sarcoma patients.

There are myriad clinical trials, both completed and ongoing, that explore the use of Dis treatment in various neoplasia including non-small cell lung cancer (NSCLC), hepatocellular carcinoma, glioblastoma, multiple myeloma, pancreatic, prostate, and metastatic breast adenocarcinomas [53–63]. Dis in conjunction with chemotherapy demonstrated a significant overall survival benefit for patients with metastatic NSCLC compared with patients treated with chemotherapy alone [62]. In a Phase 1 trial of Dis and Cu alone in the management of metastatic hepatic tumors, none of the subjects displayed any partial or complete responses, supporting the need for additional cytotoxic agents [63]. Additionally, there was a similar synergistic effect due to sensitization when the cells were introduced to radiotherapy and when all 4 treatment modalities were combined (Dis, Cu, ؤisplatin, and radiation). This provides further evidence that supports the use of Dis + Cu and compels further investigation through clinical trials focused on a combination of Dis, Cu, and conventional chemotherapy in the treatment of refractory sarcoma. To date, there have been no clinical trials focused on sarcomas or pediatric cancers using Dis.

There are several limitations to this study that must be noted. The study has been performed over several years, and both LM2 and LM7 cells have been used by our group at different times in different experiments. While this is hardly ideal, we suggest that the reader interpret these data from the lens of comparison between a low metastatic human OS cell line (SaOS2) and a related but more metastatic variant (LM2 or LM7). From this point of view, LM2 and LM7 may be considered interchangeable. One might have expected LM7 to be more susceptible to combination therapy including Dis, but Figure 8 did not bear this out. Indeed, our previous work [17] also demonstrated that despite differences in *ALDH* expression and activity between murine OS cells of differing metastatic potentials, “triple treatment” with combined Dox, Dis, and Cu displayed activity in both populations. We feel that the most important take-home message of this experiment was to demonstrate that “triple treatment” produced the most dramatic and sustained elimination of human OS cell viability. These data also serve to remind us that ALDH is likely only a part of the mystery behind the factors, pathways, and processes that drive OS metastatic potential.

It is also reasonable to question why additional human OS cells lines were not employed to demonstrate differences in Dis toxicity between MSCs and human OS cells as shown in Figure 4. Our group previously demonstrated that Dis treatment diminishes the viability and metastatic behavior of the human OS cell lines SaOS2 and SJSA [37] and K7M2 mouse OS cells [17], so we did not feel that additional cell lines were necessary to make this point. Due to the rarity of sarcomas compared with carcinomas, there is an inherent paucity of human clinical samples to study. Although we found statistically significant differences in *ALDH* gene expression and Cu levels in our patient samples, we acknowledge that our sample size is limited and includes other histologies besides OS in the experiments that analyzed Cu levels in the blood plasma of humans and canines. This may be viewed as both a limitation and a strength. Certainly, a pure population of many OSs with both metastatic and nonmetastatic clinical histories would have been ideal. Alternatively, perhaps the addition of other sarcoma histologies suggests a conserved aspect of sarcoma metastatic biology. This is an area that we tend to explore more thoroughly and invite others to do the same. Currently, we have not identified the biologic mechanism behind the aberrant Cu levels observed between low and highly metastatic OS or the mechanism by which this facilitates metastasis. We will continue to study these phenomena and leverage these data to advance the repurposing of Dis + Cu for treatment-refractory sarcoma in human patients.

## 5. Conclusions

These experiments demonstrate concordance with our prior observations that *ALDH* gene expression and Cu concentration are directly and inversely related to OS metastatic behavior, respectively. The feasibility and rationale of combination therapy with Dox, Dis, and Cu have been demonstrated in human OS cells as the only *in vitro* treatment we evaluated that durably eliminated OS cells with high metastatic potential. Based on these data, we submit that treatment with Dox, Dis, and Cu should be a considered as a viable strategy for future early phase trials for sarcoma patients with metastatic and treatment refractory disease.

## Data Availability

The Cu level, qPCR, and cell viability data used to support the findings of this study are available from the corresponding author upon request.
